# Implications of Serum 25-Hydroxyvitamin D on the Prevalence of Neoplastic Polyps: A Cross-Sectional Study

**DOI:** 10.4021/gr291e

**Published:** 2011-03-20

**Authors:** Charlene A. LePane, Gurpreet Singh, Jennifer A. Spanier-Stiasny, David M. Svinarich, Ronald J. Rasansky, Stephen M.J. Hoffman

**Affiliations:** a Department of Gastroenterology, St. John Providence Health System, Madison Heights, MI 48071, USA; b Department of Research, Providence Hospital, Southfield, MI 48075, USA

**Keywords:** 25-Hydroxyvitamin D, Neoplastic polyps, Colonoscopy

## Abstract

**Background:**

Vitamin D is believed to help in the suppression of malignant cells. Epidemiologic studies suggest that there is an association between vitamin D deficiency and an increased risk of colorectal cancer. The primary aim of this study is to determine if the prevalence of neoplastic polyps is inversely related to serum 25-hydroxyvitamin D levels 25(OH)D.

**Methods:**

A prevalence study conducted between April 2009 and October 2009 evaluated 651 patients undergoing colonoscopy in order to determine if an association existed between low 25(OH)D levels and the prevalence of neoplastic colon polyps. Multivariate logistic and linear regression analyses were used to establish an association between 25(OH)D levels and histology of colon polyp with gender, race, age and BMI.

**Results:**

The presence of tubular adenoma, villous adenoma, tubulo-villous adenoma, or malignancies did not differ (P = 0.5) among the stratified 25(OH)D groups (10 ng, 10.1 - 30 ng, > 30 ng). In addition, despite having more African-Americans than Caucasians in the lowest 25(OH)D category (22.7% versus 7.7%), the presence of neoplastic polyps did not differ significantly (P = 0.8) between the categorized racial groups (Caucasian and African-Americans).

**Conclusions:**

Low plasma 25(OH)D levels are not associated with an increased prevalence of neoplastic polyps.

## Introduction

Vitamin D has been reported to decrease the risk of colorectal cancer through various mechanisms, including reducing cell proliferation, inhibiting angiogenesis, promoting cell differentiation and stimulating apoptosis [1]. The exact mechanism remains under current study. It was the increased occurrence of colorectal cancer (CRC) at higher latitudes that prompted investigation of vitamin D as a chemoprotective agent. In 1980, Garland et al. reported an inverse relationship between observed risks of colorectal cancer and stratified dietary vitamin D-calcium index. Subsequently, multiple studies have been carried out to investigate the relationship between vitamin D and adenoma (precursor lesion to CRC) prevalence. A chronological summary of the literature describing the above relationship is as follows: Kampman et al. (1994) enrolled patients from the Health Professionals Follow-up Study, and the Nurses’ Health Study, who underwent sigmoidoscopy. No relationship was observed between total vitamin D intake and the risk of left-sided adenoma in men. However, there was a slight, but not significant inverse association between vitamin D intake and risk of adenoma in women. Platz et al. (2000) also enrolled patients from the Nurses’ Health Study (326 Cases, 326 Controls) and associated plasma 25(OH)D to be inversely related to distal colorectal adenoma; oddly, the benefit of vitamin D on polyp occurrence was not observed in patients belonging to the highest plasma 25(OH)D quartile. Grau et al. (2003) correlated high serum 25(OH)D levels with reduced adenoma risk only among subjects receiving calcium supplements; this study enrolled high risk individuals from the polyp prevention study. Jacobs et al. (2007) enrolled high risk individuals from the Ursodeoxycholic Acid trial; and concluded that there was a nonsignificant inverse association between serum 25(OH)D and adenoma recurrence, particularly among women. Our primary aim was to explore whether high serum 25(OH)D offered a chemoprotective effect on adenoma occurrence in average risk individuals.

## Mmaterials and Methods

### Patient population

Our study information was submitted to the St. John Providence Hospital and Medical Center Institutional Review Board and was approved with a designated study number of 09127 on April 17, 2009. Patients were then consecutively enrolled by a nurse trained in the study protocol at a community hospital open endoscopy unit from April 2009 to October 2009. A Serum 25(OH)D level was drawn upon patient consent for enrollment in the study. The endoscopist was blinded to the enrolled patients and 25(OH)D levels. Among the 726 patients admitted to the endoscopy unit for colonoscopy, 651 patients were enrolled after exclusion criteria were taken into account. The study included adults age 18 years or older, regardless of gender or ethnicity, who demonstrated a willingness to participate in the study. Exclusion criteria included an inability to visualize the entire colon, history of inflammatory bowel disease, Boston bowel preparation score less than six, or previous history of polyps. Patients undergoing colonoscopy did not receive compensation.

### Questionnaire, laboratory assays and neoplastic polyp evaluation

Information obtained included the primary indication for colonoscopy, age, gender, BMI and ethnicity. Patients were questioned about known history of previous polyps, previous diagnosis of colon cancer, previous colonoscopy with year and results, if applicable. Additionally, the patients’ use of multivitamin and or vitamin D supplementation with the frequency, dose, and duration were documented. Prior to undergoing colonoscopy, participants’ serum 25(OH)D levels were determined. At the completion of the procedure the Boston bowel preparation score, removal time, and whether the entire colon was visualized were documented. Among participants, all adenomatous and malignant polyps were removed and directly sent to pathology. All lab and pathology specimens were processed and evaluated at St. John Providence Macomb-Oakland Hospital, Oakland Center.

### Data analysis

The primary aim of this study was to test the null hypothesis that the increased prevalence of neoplastic polyps was inversely related to serum 25-hydroxy Vitamin D levels. Multivariate logistic and linear regression analyses were used to establish an association between 25(OH)D levels and histology of colon polyps with gender, ethnicity, age and BMI. Population studies demonstrate that approximately 30% of middle-aged to elderly individuals possess polyps. Adenomatous polyps constitute about 10% of all polyps. The percentage of polyps obtained throughout this study was 63%. The criterion for significance (alpha) was set at 0.050. Statistical significance was set at P < 0.05. Statistical calculations were made using the ANOVA program.

## Results

### Recruitment and participant flow

Between the 17th of April 2009, and the 26th of October 2009, patients who were referred to our endoscopy center were provided with a detailed description of our study and the opportunity to enroll. If the patient agreed to participate, a screening questionnaire was provided to the patient along with a trained nurse to assist with questions. All patients had the opportunity to ask questions regarding the study prior to signing an informed consent for blood draw. There were three exclusion criteria: entire colon not visualized, patient history of inflammatory bowel disease (ulcerative colitis or Crohns) or a Boston bowel prep score of less than 5. A total of 726 patients were enrolled in the study. Eight patients were excluded secondary to history of inflammatory bowel disease. An additional 42 patients were excluded due to a low Boston bowel prep score, and 25 patients were excluded because the entire colon was not visualized.

### Patient demographics

A total of 651 patients were included in the study. Of these, 429 patients (65.9%) were Caucasian, 128 patients (19.7%) were black and 94 patients (14.4%) were of other ethnicity (Hispanic, Asian, European, and Middle Eastern). The cohort consisted of 324 (49.7%) males and 327 (50.3%) females. Within the cohort 22 (3.9%) patients were under the age of 30 years, 410 (62.9%) patients were between the ages of 31 - 60 years and 219 (33.6%) patients were greater than the 60 years of age (mean age 55.9 and range 17 - 92).

### Colonoscopy details

Indications for colonoscopy for the 651 participants included routine screening 245 (37.6%), previous GI bleed 136 (20.9%), polyp surveillance 87 (13.4%), family history of colorectal cancer 11 (1.7%), anemia 34 (5.2%), abdominal pain 29 (4.5%), weight loss 6 (0.9%), diarrhea 38 (5.8%), constipation 30 (4.6%), abnormal imaging 10 (1.5%), malignancy 10 (1.5%), and colitis 15 (2.3%). Among the cohort population 128 subjects (19.7%) had a previous colonoscopy, whereas 523 subjects (80.3%) underwent their first colonoscopy. There were 5 trained endoscopists performing colonoscopies on study subjects. All patients were prepped for colonoscopy by ingestion of one gallon of Golytely the evening before the procedure. An Olympus CF-H180 AL/I colonoscope was used on all patients. Narrow band imaging (NBI) and magnification was available for the endoscopist as an accessory tool to evaluate polyps. The minimum size of polyp for polypectomy was 3 mm. Polypectomy with or without electrocautery was performed with Boston Scientific Profile, Captiflex or Captivator II snares. Withdrawal times were documented for each colonoscopy providing a mean time of 9.63 minutes (range 6 - 60 minutes). Evaluation of the cecum with or without terminal ileum intubation constituted complete visualization. The Boston bowel preparation scale was used as a reliable measure of bowel preparation with a score of 0 - 5 indicating inadequate prep and 6 - 9 representing adequate prep [2].

### Polyp analysis

**Figure 1:**
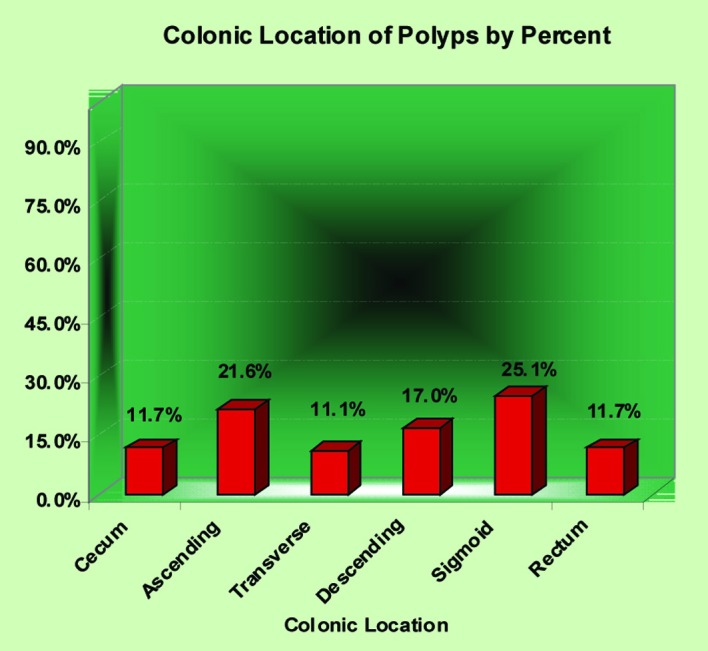
Colonic location of polyps by percent.

All polyps removed were histologically examined by the institutional site pathologist employing the diagnostic criteria defined by the National Polyp Study [3]. Among the 651 subjects studied, 480 (73.7%) did not have polyps and 171 (26.3%) had at least one polyp detected by colonoscopy. For the 171 subjects with polyps, the exact location of removal with the histological finding of that polyp was recorded. Three subjects that did not have the exact location of polypectomy entered in our data analysis therefore analysis of particular location with subsequent histological evaluation was for 168 total polyps. Twenty polyps (11.9%) were removed from the cecum, 37 (22.0%) from the ascending colon, 19 (11.3%) from the transverse colon, 29 (17.3%) from the descending colon, 43 (25.6%) from the sigmoid colon and 20 (11.9%) from the rectum (Fig. 1).

Because we ascertained the presence of hyperplastic and adenomatous polyps in our study population, we were able to stratify polyps into two groups; cases with non-adenomatous polyps and cases with adenomatous polyps. The histological findings of these polyps were then analyzed statistically for each anatomical location of the colon (Table 1). Note that “other” was defined as indeterminant.

**Table 1:** Histological Classification of Polyps Evaluated for Colon Location

	All	Cecum	Ascending	Transverse	Descending	Sigmoid	Rectum
	N (%)	N (%)	N (%)	N (%)	N (%)	N (%)	N (%)
Hyperplastic	50 (29.8)	2 (10)	13 (35.2)	5 (26.3)	7 (24.1)	18 (41.9)	5 (25)
Tubular Adenoma	97 (57.7)	13 (65)	18 (48.6)	14 (73.7)	18 (62.1)	23 (53.5)	11 (55)
Villous Adenoma	1 (0.6)	1 (5)	0 (0)	0 (0)	0 (0)	0 (0)	0 (0)
Tubulovillous Adenoma	8 (4.7)	0 (0)	4 (10.8)	0 (0)	0 (0)	2 (4.6)	2 (10)
Malignant	6 (3.6)	3 (15)	0 (0)	0 (0)	1 (3.4)	0 (0)	2 (10)
Other	6 (3.6)	1 (5)	2 (5.4)	0 (0)	3 (10.4)	0 (0)	0 (0)
Totals	168 (100)	20 (11.9)	37 (22)	19 (11.3)	29 (17.3)	43 (25.6)	20 (11.9)

### Vitamin D analysis

Serum 25(OH) vitamin D levels were drawn from each subject prior to colonoscopy. All results were provided by the institutional lab, which defines vitamin D deficiency as < 30 ng/ml. The normal range of 25(OH) vitamin D is 30 ng/ml - 74 ng/ml. Vitamin D supplementation and details of frequency, length of time and dose were assessed on the screening questionnaire. A total of 104 (16%) patients out of 651 patients were currently on vitamin D supplements. Of those, 93 patients (89.4%) took vitamin D daily, 7 patients (6.7%) took vitamin D once weekly and 4 patients (3.8%) did not take vitamin D on a regular basis. The range of intake duration was from 1 month to 180 months with a mean of 22 months. Of the 104 subjects taking vitamin D supplements, 34 (32.7%) were unsure of dose, 16 (15.4%) took 400 IU in a standard multivitamin, 18 (17.3%) took 1000 IU dosage, 7 (6.7%) took 2000 IU dosage, 2 (1.9%) took 5,000 IU dosage and 27 subjects (26%) documented “other” dose of vitamin D. 25(OH) vitamin D levels of all 651 subjects were stratified as severely deficient (< 10 ng/ml), deficient (10.1 ng/ml - 30 ng/ml), and therapeutic (> 30 ng/ml). Seventy-two patients (11.1%) were severely deficient, 407 patients (62.5%) were deficient, and 172 (26.4%) were therapeutic. The mean 25(OH) vitamin D level was 23.9 ng/ml (range 4 - 75 ng/ml).

### Outcomes

The data was analyzed by ANOVA initially with all 651 subjects and subsequently with only 547 subjects not taking vitamin D supplementation currently. Multivariate logistic and linear regression analyses were used to establish an association between 25(OH) vitamin D with gender, age, and race (Table 2). In a *post hoc* analysis, the Pearson Chi-Square coefficient did not demonstrate a statistically significant relationship between 25(OH) vitamin D levels and the presence of polyps in the 651 subjects (P = 0.739 CI 95%). In addition, there was no statistically significant relationship between 25(OH) vitamin D levels and the 547 subjects who were not on vitamin D supplements (P = 0.709 CI 95%) (Table 3). Furthermore, there was no statistically significant relationship between 25(OH) vitamin D level and presence of adenoma in either the 651 subject analysis (P = 0.487 CI 95%) and the 547 subject analysis (P = 0.525 CI 95%) (Table 4).

**Table 2:** Multivariate Analyses for Polyps, Adenomas, and 25(OH) Vitamin D Levels Adjusted for Race, Gender and Age

	Polyps		P Value	Adenoma		P Value	25(OH) vit-D			P Value
	N	Y		N	Y		< 10	10.1 - 30	> 30	
	Number (%)	Number (%)		Number (%)	Number (%)		ng/ml	ng/ml	ng/ml	
Race			0.541			0.816				0.000 (95%) CI
Caucasian	311 (72.5)	118 (27.5)		348 (81.1)	81 (18.9)		33 (7.7)	253 (59.0)	143 (33.3)	
Black	99 (77.3)	29 (22.7)		105 (82.0)	23 (18.0)		29 (22.7)	84 (65.6)	15 (11.7)	
Other	70 (74.5)	24 (25.5)		74 (78.7)	20 (21.3)		10 (10.6)	70 (74.5)	14 (14.9)	
Gender			0.001 (95%) CI			0.009 (95%) CI				0.225 (95%) CI
Male	220 (67.9)	104 (32.1)		249 (76.9)	75 (23.1)		34 (10.5)	213 (65.7)	77 (23.8)	
Female	260 (79.5)	67 (20.5)		278 (85)	49 (15)		38 (11.6)	194 (59.3)	95 (29.1)	
Age			0.000 (95%) CI			0.001 (95%) CI				0.800 (95%) CI
< 30 yo	21 (95.5)	1 (4.5)		21 (95.5)	1 (4.5)		1 (4.5)	16 (72.7)	5 (22.7)	
31 - 60 yo	319 (77.8)	91 (22.2)		345 (84.1)	65 (15.9)		48 (11.7)	255 (62.2)	107 (26.1)	
> 60 yo	140 (63.9)	79 (36.1)		161 (73.5)	58 (26.5)		23 (10.5)	136 (62.1)	60 (27.4)	

**Table 3:** Relationship Between 25(OH) Vitamin D and Presence of Polyps

25(OH) vit D	Polyp N	Polyp Y	Total	P Value
	Number (%)	Number (%)	Number	(Chi-Square)
< 10 ng/ml	54 (75.0)	18 (25.0)	72	
10.1 - 30 ng/ml	303 (74.4)	104 (25.6)	407	
> 30 ng/ml	123 (71.5)	49 (28.5)	172	
Totals	480 (73.7)	171 (26.3)	651	0.739

**Table 4:** Relationship Between 25(OH) Vitamin D and Adenoma

25(OH) vit D	Adenoma N	Adenoma Y	Total	P Value
	Number (%)	Number (%)	Number	(Chi-Square)
< 10 ng/ml	62 (86.1)	10 (13.9)	72	
10.1 - 30 ng/ml	326 (80.1)	81 (19.9)	407	
> 30 ng/ml	139 (80.8)	33 (19.2)	172	
Totals	527 (81.0)	124 (19.0)	651	487

## Discussion

In this prospective cross-sectional study, we found no statistically significant association between 25(OH) vitamin D levels and occurrence of polyps, both adenomatous and non-adenomatous.

One of the first studies in the literature to report a relationship between vitamin D and colon cancer was by Garland et al in 1980 [4]. They found that reduced solar ultraviolet-B radiation exposure in turn resulted in lower serum levels of vitamin D which could account for the increase in mortality from colon cancer among individuals who resided at higher altitudes. Several studies since have suggested an inverse relationship between 25(OH) vitamin D and body mass index, blood pressure and diabetes [5-8]. Vitamin D has been postulated in several studies to be an anticarcinogenic nutrient by preventing development of colorectal cancer [9, 10]. A recent case-control study by Wu et al. demonstrated a statistically significant inverse association between 25-hydroxyvitamin D concentration and colon cancer. However, in the same study there was a non-statistically significant relationship found with colorectal cancer, thus implicating its protective effect on the colon only and not in the rectum [11]. After the Women’s Health Initiative demonstrated that low dose vitamin D did not protect against colorectal cancer, a quantitative meta-analysis study by Gorham et al. proposed that a higher dose might be protective [12]. The pooled results of five serum 25(OH) vitamin D studies were divided into quintiles and an odds ratio was formulated. A 50% lower risk of colorectal cancer was associated with a serum 25(OH) vitamin D level ≥ 33 ng/mL, compared to ≤ 12 ng/mL. This evidence suggests that daily intake of vitamin D 1000-2000 IU/day may possibly reduce the incidence of colorectal cancer. A more recent meta analysis by Yin et al. analyzed eight previous longitudinal studies of serum vitamin D levels and colorectal cancer risk and suggested that vitamin D is inversely related to CRC risk [13].

Although low vitamin D status has long been implicated in colorectal carcinogenesis, we thoroughly investigated the literature after our study findings and have not been able to discern a clear relationship that is consistent with anticarcinogenic effects of vitamin D on colorectal cancer. We found several well-designed studies that have elicited findings that oppose the previous thought that vitamin D is inversely related to colorectal carcinoma. As mentioned previously Morimoto et al. examined diet and lifestyle risk factors for hyperplastic and adenomatous polyps [14]. They found calcium intake > 1275 mg/day to be protective against hyperplastic polyps, but not adenomatous polyps. In a multivariate analysis of 794 patients with polyps (adenomatous and non-adenomatous types), they found that intakes of other dietary nutrients (fat, folate, vitamin D and multivitamins) were not associated with polyp risk of any type. Platz et al. evaluated plasma 1,25-dihydroxy- and 25(OH) vitamin D and adenomatous polyps of the distal colorectum [15]. Their cases and controls were drawn from among participants in the Nurses’ Health Study and they concluded that women who have low levels (< 26 pg/dL) of circulating 1,25(OH)2D may be at higher risk of distal colorectal adenomas only. This finding is consistent with the outcome of the Nurses’ Health Study, a cohort of 121,700 women as well as 50,000 men, in which there was no association between vitamin D intake from diet of supplementation and colorectal adenoma was found. There was, however, a statistically significant relationship found between vitamin D and rectal adenoma in women. This finding challenges the previous findings by Wu et al. in which vitamin D had no protective effect on rectal adenomas. Kampman et al. evaluated calcium, vitamin D, dairy foods and the occurrence of colorectal adenomas in 2 prospective studies [16]. The cases were patients with adenomatous polyps found in the left colon or rectum, and controls were persons with endoscopic findings negative for adenoma. They concluded that the occurrence of colorectal adenoma was neither related to calcium intake nor to milk consumption, whereas vitamin D from supplements but not diet was slightly, but not significantly, inversely associated with risk among women only. In a retrospective analysis of colon cancer and serum vitamin D metabolite levels 10 - 17 years prior to diagnosis of colon cancer by Braun et al. both 1,25(OH)2D and 25(OH)D were evaluated [17]. Their data provided no strong support for the hypothesis that vitamin D metabolite levels affect the subsequent risk of colon cancer. In a nestled case-control study of Finnish men by Helzlsouer et al. cases were participants diagnosed with primary adenocarcinoma of the large bowel [18]. Pre-diagnostic serum levels of both vitamin D metabolites (1,25(OH)2D and 25(OH)D) were used as primary exposure measures. No association was seen between serum levels of 1,25(OH)2 vitamin D and large bowel cancer risk. However, the estimated relative risk (RR) of large bowel cancer decreased with increasing levels of serum 25(OH)D and the relationship was more pronounced for rectal cancer. Thus, they demonstrated no evidence of effect modification by level of 1,25(OH)2D for large bowel cancer, but once again demonstrated that high levels of circulating 25(OH)D may be protective against rectal adenocarcinoma consistent with other studies mentioned above. Using data from a case-control study conducted in 1994 in Northern Italy, Ferraroni et al. analyzed the relationship between estimated intake of micronutrients and the risk of colorectal cancer [19]. They found no apparent trend in risk across intake quintiles of retinol, vitamin D, methionine and calcium. Interestingly, they did find a trend of a protective effect with increasing consumption of beta carotene, ascorbic acid, vitamin E and folic acid. It was this study, among several others that prompted the extensive evaluation of the role of folic acid in colorectal cancer prevention at that time, analogous to the extensive study of vitamin D now in CRC prevention.

As mentioned previously, the Women’s Health Initiative results contributed significantly to the vitamin D and neoplasia literature [20]. Watctawski-Wende et al. organized a randomized, double-blind placebo-controlled trial of 36,282 women who were given a supplement of 500 mg of calcium carbonate plus 200 IU of vitamin D3 twice daily, or a placebo. The results demonstrated that daily supplementation of calcium with vitamin D for seven years had no effect on the incidence of colorectal cancer among postmenopausal women. This finding prompted the subsequent nested case-control analysis by Wu et al. previously noted. Jacobs et al. utilized study participants from the Ursodeosycholic Acid Trial in attempt to assess the relationship between serum 25(OH) D levels, dietary intake of vitamin D, and colorectal adenoma recurrence [21]. Analyses by dietary vitamin D intake revealed no statistically significant associations. These findings showed a moderate, nonsignificant inverse association between serum 25(OH)D levels and reduced risk for colorectal adenoma recurrence, particularly among women. In a multi-center, placebo-controlled randomized clinical trial by Grau et al. the independent and joint effects of calcium supplementation and vitamin D status on adenoma recurrence were evaluated [22]. They concluded that calcium supplementation and vitamin D status seemed to act largely together, not separately, to reduce the risk of colorectal adenoma recurrence. Additionally, they found that vitamin D receptor (VDR) polymorphisms were not related to adenoma recurrence and did not modify the associations with vitamin D or calcium. There appears to be some conflict in the literature regarding VDR relationship, as it has been studied to be associated with various cancers, including colorectal carcinoma [23].

### Strengths of the study

Evaluation of the precise delineation of polyp borders and advanced macroscopic analysis of all polyps were performed with the optional application of narrow band imaging (NBI) and magnification. Previous studies have demonstrated no statistically significant benefit of NBI with polyp detection [24, 25]. However, a recent study supports increased polyp detection and histologic correlation with NBI, therefore suggesting that this technique be considered by endoscopists in order to optimize polyp detection rate [26]. Possible confounding factors were avoided in this study due to the consistent timing of and ingested amount of prep (1 gallon Golytely), the minimal size of polyp to be removed (≥ 3 mm), minimum accepted withdrawal time of 6 minutes, mean of bowel prep scores ≤ 5 and patients with inflammatory bowel disease given the association with increased risk of colorectal carcinoma [27]. There was no selection bias in our study because we accepted subjects of any age, sex or gender who were to undergo colonoscopy. Our study demonstrated a detection rate of adenomas in men to be 23.1% and females 15.0%, which is consistent with the current recommendations of the U.S. Multi-Society Task Force in patients above the age of 50 [28].

### Limitations of the study

There were a number of limitations in our study. First, we did not account for aspirin intake which has been shown to be protective against colorectal cancers that overexpress COX-2 [29]. We also did not stratify for smoking status which may be a potential limitation as it is a widely accepted risk factor for development of neoplastic colonic polyps. Given a study population of 651 subjects, only 124 subjects had adenomatous polyps which reflects a relatively small sample size. Therefore, it is possible that some associations in our study were masked because of insufficient power. Despite advances in polyp detection due to the advent of NBI and magnifying endoscopy, there remains a miss rate of 15% - 25% of neoplastic polyps < 1 cm and 0% - 6% of those larger than 1 cm based on studies of tandem colonoscopies [30]. Although malignant potential is low for small polyps it is reasonable to suggest that polyps < 3mm that were not removed in this study possessed neoplastic potential and are unaccounted for in our study. We did not assess for liver, kidney or parathyroid disease which would provide lower than normal vitamin D levels. There may be variations in diet (i.e., diet fortified with Vitamin D) that were also not accounted for which may have elevated 25(OH) Vitamin D in some individuals. Furthermore, we did not screen for family history of familial adenomatous polyposis (one case for every 6580 - 8300 persons) or hereditary nonpolyposis colorectal cancer based on a lack of genetic testing. Our study may be limited by its clinic-based setting; because only patients who underwent colonoscopy were eligible for this study and this population certainly may not represent the general population. Because season of the year in which blood was collected likely influences levels of 25 (OH) Vitamin D, this may have confounded our data because we did not repeat multivariate analysis using separate cutpoints for season of blood collection. Additionally, a measurement error in circulating Vitamin D metabolite concentration is plausible given that we measured these levels at one particular point in time. Lastly, despite noting the 3 mm cut-off for polypectomy, we did not document the size of the polyp which should prompt future investigation for correlation with Vitamin D levels.

In summary, although there were 106,100 new cases or colon cancer and 40,870 cases of rectal cancer in 2009 with a reported 49,920 deaths from both, colorectal cancer remains one of the most preventable malignancies [31]. It has been estimated that approximately 70% of CRC could be avoided by dietary modifications, in particular, supplementation of vitamin D. Epidemiological and clinical researchers have sought to investigate this proposed relationship between vitamin D and colorectal carcinoma. To date, there have been several studies that report a nonsignificant inverse relationship between vitamin D and colorectal malignancies, while many others have not been shown to prove this theory.

Our study did not provide a statistically significant relationship between 25(OH) vitamin D and adenomatous polyps in average risk individuals. A follow-up meta analysis of all studies in support of our findings should be pursued in an attempt to establish such a relationship in light of the conflicting data pertaining to what role of vitamin D plays in the prevention of colon polyps and colorectal cancer. There is currently a government-supported large patient population study that is investigating the effect of vitamin D on a panel of biomarkers of risk for colorectal cancer. Additionally, the National Cancer Institute is supporting a phase I randomized control trial evaluating vitamin D supplementation in prevention of colon cancer in African Americans with colon polyps. These studies in conjunction with other ongoing trials hold potential to clarify the previous conflicting data with regards to the relationship between vitamin D and neoplasms of the colon. Until then, the role of vitamin D in prevention of adenomatous polyps and colorectal cancer remains controversial and future studies are warranted.
